# Aligning Roles and Responsibilities: Perspectives of Medical Students and Supervisors in Undergraduate Research

**DOI:** 10.1007/s40670-026-02656-0

**Published:** 2026-03-02

**Authors:** Melissa Shiff, Erik Montagna, Colm O’Tuathaigh

**Affiliations:** 1 School of Medicine, University College Cork, Cork, Ireland; 2 Faculdade de Medicina do ABC, Centro Universitário FMABC, Santo André, SP Brazil; 3 Medical Education Unit, School of Medicine, University College Cork, Cork, Ireland

**Keywords:** Research-based learning, Medical students, Supervision, Q-methodology

## Abstract

Active participation of medical students in research activities enables their understanding of research methods and the benefits that research can provide them as both researchers and clinicians. Differing expectations on the part of the student and supervisor regarding their relative roles and expected contribution can influence the achievement of learning outcomes and quality of the research project. Here we explore the expectations of medical students and supervisors regarding the roles and responsibilities of both parties during the completion of an undergraduate medical research project. Employing a mixed methods design, the CREDIT URE tool, which defines, and measures roles performed by undergraduate students working in research placements, was completed by 226 medical students and their project supervisors. Q-methodology was then used to examine perspectives of students and supervisors on their relative responsibilities. Q-set data were analysed using a by-person factor analysis to group participants with shared viewpoints. Using the CREDIT URE tool, students and their project supervisors rated writing of the original draft article, visualisation, data curation, formal analysis, and investigation as the most common roles and those with the highest level of student responsibility. In the Q methodology study, a five-factor solution (i.e. profiles) provided the best fit for the data collected from 25 participants (16 student, 9 supervisors), explaining 55% of the variance. Each profile describes a shared viewpoint on project roles. We characterized the profiles as ‘student-driven’, ‘supervisor as guide and overseer’, ‘self-motivated student’, ‘supervisor involved only as needed – skills development’ and ‘supervisor as editor’. Medical schools increasingly emphasise research competencies as part of undergraduate education. These data are expected to lead to the development of clear supervision frameworks and role descriptions, and by extension may inform the design of undergraduate medical research modules or project guidelines.

**Supplementary Information:**

The online version contains supplementary material available at 10.1007/s40670-026-02656-0.

## Introduction

Accrediting bodies worldwide have highlighted the need for the development of students’ research competencies during undergraduate medical education [1–5]. Participation in research at an undergraduate level promotes the development of transferable skills that are relevant to medical graduates who wish to pursue an academically orientated research role as well as a clinical practice-orientated career [6–8]. Encouragement of active participation in research also encourages a better understanding of how research may contribute to quality of patient care and is critical for the development of clinician-academics. Medical schools worldwide use a wide variety of strategies to address these educational goals; these strategies range from performing scientific literature reviews of clinically related topics during placements, to completing in-depth, independent research projects [6, 9]. Due to resource scarcity, some programs have chosen to focus effort and resources on a smaller group of students and encourage them to obtain advanced degrees. Other programmes promote a more inclusive approach based on encouraging the development of research competencies in a larger cohort [7]. Congruent with theories of communities of practice [10], programmes generally encourage authentic research problems within existing clinical or research teams.

Participation in research programs which prioritise active participation promotes research skills including formulation of clinical research questions, literature search, critical appraisal, data analysis and interpretation, as well as soft skills including teamwork, communication, and presentation skills, all of which are core learning outcomes listed by global standards for medical education [8, 9, 11]. Previous studies have supported an association between immersive research experiences during medical school and tangible research outputs engendered (including publications and presentations), as well as achievement of research- and evidence-based medicine-related learning outcomes (see [12] for review).

Some authors have questioned the benefits to the student and medical schools of undergraduate research participation [13]. Some of these concerns hinge on pragmatic concerns including costs, supervisor availability, and how such opportunities are provided within what is often a crowded curriculum [e.g. 6]. Some have additionally highlighted the difficulty for transitioning from coursework to independent research [14]. Others have questioned student motivation for doing research, where it might be overly focused on accumulation of low impact research outputs to increase competitiveness of residency applications [e.g. 15].

A variety of models for integrating research experience into the undergraduate medical curriculum have been described and implemented [6]. The most intensive approach involves students completing an extended research project under the supervision of an academic and/or clinical staff member, typically culminating in submission of a written dissertation. At University College Cork, the School of Medicine offers active, experiential, project-based learning experience, where students are required to develop these skills and competencies through participation in independent research under the supervision of academic and clinical staff. In addition to this systemic and mandatory uniform experience, many students engaged in ongoing research projects, both with institutional faculty members and through placements with national and international research groups during non-curricular time [16, 17].

It has been noted that many academics’ or clinicians’ first research supervisory experience is with undergraduate projects and in many cases, this will not involve formal supervisory training but rather learning through experience [18]. Many medical project supervisors are additionally required to balance the demands of supervision with their existing clinical workload. The student-supervisor relationship has been cited as a key determinant of student satisfaction with the research experience, as well as achievement of learning outcomes [19]. Studies have shown differing expectations on the part of the student and supervisor regarding their relative roles and expected contribution [20, 21]. For example, while supervisors expect to provide a more ‘big picture’, less practically-focused supervision which enhances students’ independence in training, students can often expect more support with data collection and analysis, as well as more specific direction and feedback during preparation of deliverables [21].

Many of these studies have come from various disciplines but not in a medical education context, and so the first aim of the present study was to develop a better understanding of the student and their supervisor’s perceptions of contributions made by students during their completion of an undergraduate medical research project. Using Q-methodology, the second aim of this study was to explore patterns in relation to students’ and supervisors’ understanding of the relative contribution of both parties to the undergraduate research project. Q-methodology is a mixed-methods approach which is used to systematically identify and compare subjective viewpoints on a given topic. Participants sort a set of statements along a forced distribution based on agreement or relevance, and factor analysis is then used to group participants with shared perspectives. This method is increasingly applied in health professions education to characterise divergent beliefs and role expectations [22]. Based on these two components, we discuss the implications and potential approaches to addressing divergent views around role expectations during the undergraduate project.

## Methods

### Setting

University College Cork offers both a 5-year direct-entry medicine (available to students who have completed secondary education) and a 4-year graduate entry medicine programme. Students are required to complete an independent research project (see [16]). The scope of projects is diverse and includes traditional clinical research (across specialties), laboratory-based biomedical projects, public health, and medical education. From initial conceptualization of the project idea to submission of the dissertation, the project is completed over a two-year period between years 3–5 of direct-entry medicine (years 2–4 of the graduate entry curriculum). To contextualise this activity, these students commence clinical clerkships during year 3 of the direct-entry programme (year 2 of graduate-entry programme). A number of checkpoints are introduced to ensure that timely progress is achieved; these include submission of a project proposal (including a literature review), project progress updates via oral presentation, application for research ethics approval, oral presentation of research results at a dedicated symposium, submission of a research paper (5,000 words). Students are supervised individually by full-time academic and/or consultant-grade physicians from across the network of University College Cork teaching hospitals. Prospective supervisors are provided with materials describing the programmes and resources available, as well as links to project guidelines (including student and supervisor descriptions; Supplementary Data 1) and University research policies but no formal training is provided otherwise. School funding is not available to support all individual projects. Dimensions of undergraduate research and inquiry are embedded throughout the programme; this is achieved by provision of stage-appropriate research teaching by School academic staff in a structured and deliberate manner across the lifetime of the project. This study was approved by the Social Research Ethics Committee of University College Cork.

### Contributor Roles Taxonomy (CREDIT) URE

Here we employ the Contributor Roles Taxonomy (CREDIT) Undergraduate Research Experience (URE) tool, adapted by Honore et al. [23] to collect and record high-level classification of the diverse roles performed during the project. Originally designed to bring transparency around research contribution, attribution, and authorship credit [24], the taxonomy describes the following 14 roles: conceptualisation, data curation, formal analysis, funding acquisition, investigation, methodology, project administration, resources, software, supervision, validation, visualization, writing – original draft, writing – reviewing and editing. Two parallel instruments were used: one for students and one for their supervisors to complete. For each of the roles, the respondent (student or supervisor) was asked to specify the level of student contribution based on a Likert-style rating-scale ranging from 0 (no responsibility) to 3 (primary responsibility). Both students and their supervisors completed the CREDIT URE tool following submission of their undergraduate project paper at the end of final year of their medicine course. In the present analysis, where students are not expected to seek funding to support their undergraduate medical projects, the item which pertains to funding acquisition was omitted.

### Q-methodology

After identifying the research question, implementing Q-methodology comprises the following steps: (i) identification of study participants (p-set); (ii) elaboration of study discourse and subjective statements (Q-set); (iii) data collection by ranking the statements of the Q-set by participants (Q-sort) and iv) data analysis and interpretation. An initial pool of 101 statements was generated from a narrative review of the literature on student–supervisor role expectations and supplemented by insights from the CREDIT-URE descriptive findings. These statements were systematically reviewed by the co-authors to ensure comprehensive coverage of research governance, supervisory practices, and autonomy-related expectations. Redundant or overlapping items were removed, resulting in a final, balanced set of 99 statements (Supplementary Data 2). Participants in this study were Year 4 of the direct-entry and Year 3 graduate-entry medical students, the period during which students undertake their mandatory research project. They were recruited via a combination of convenience and snowball sampling. Snowball sampling was used to capture a presumed diversity of perspectives among students (based on academic characteristics including mode of entry to medical school, prior research experience) and project supervisors (based on clinical speciality and supervisory experience). Subsequently, participants ranked the statements from their individual perspective using a grid with a quasi-normal, forced distribution. The grid has the same number of cells as there are statements and has a rating scale from − 4 to + 4 which represents “most disagree” to “most agree.” All statements must be placed on the grid. When the ranking was completed, all responses are reduced to a few different factors (presented here as profiles), each representing a distinct group of participants, based on the analysis described in a later section. As participants in a Q-methodology study evaluate and rank a large Q-set, a limited number of well-selected participants is deemed sufficient to achieve saturation [22]. All participants completed the Q-methodology using the Q Method Software (https://qmethodsoftware.com/) platform.

This Q-methodology analysis was guided by the Research Skill Development (RSD) Framework developed by Willison and O’Regan [14]. The RSD conceptualises students’ research engagement through progressively complex levels of autonomy, highlighting how supervision structure shape students’ movement from highly guided/prescribed to increasingly self-directed inquiry.

Within this model, lower levels of autonomy involve significant supervision, where students follow prescribed research processes. Intermediate stages reflect shared control, with supervisors scaffolding decision-making as students develop research competencies. At the highest levels, students take primary responsibility for designing and executing inquiry-based projects, while supervisors principally act as facilitators, enabling students to work independently. This framework is particularly useful for undergraduate project roles because it highlights the interplay between s*tudent autonomy* and *supervision structure*.

### Data Analysis

For each of the CREDIT URE roles, descriptive statistical data for each role was calculated for students and the corresponding supervisor. The level of agreement was computed using Cohen’s kappa statistic. Additionally, responses were categorized based on substantive contribution (encompassing both moderate and primary responsibility scale scale unit responses) or otherwise (little responsibility or no responsibility). For student respondents reporting a specific role contribution (i.e. a score higher than zero), their ratings were compared with their supervisors’ ratings using the Mann-Whitney U test. Effect size(s) for Mann-Whitney U comparisons which achieve statistical significance were calculated using the rank-biserial correlation (r). The significance threshold was set at *P* < 0.05 (Table 1).

**Table 1:** The contributor roles taxonomy (CRediT), adapted from Honore et al. [22]

Role	Definition
Conceptualization	Ideas; formulation or evolution of overarching research goals and aims
Data curation	Management activities to annotate (produce metadata), clean data, and maintain research data (including software code, where necessary for interpreting the data itself) for initial use and later reuse
Formal analysis	Application of statistical, mathematical, computational, or other formal techniques to analyze or synthesize study data
Funding acquisition	Acquisition of the financial support for the project leading to publication
Investigation	Conduct of a research and investigation process, specifically performing the experiments or data/evidence collection
Methodology	Development or design of methodology; creation of models
Project administration	Management and coordination responsibility for research activity planning and execution
Resources	Provision of study materials, reagents, materials, patients, laboratory samples, animals, instrumentation, computing resources, and other analysis tools
Software	Programming, software development; designing computer programs; implementation of the computer code and supporting algorithms; testing of existing code components
Supervision	Oversight and leadership responsibility for the research activity planning and execution, including mentorship external to the core team
Validation	Verification, whether as a part of the activity or separately, of the overall replication/reproducibility of results/experiments and other research outputs
Visualization	Preparation, creation, and/or presentation of the published work, specifically visualization/data presentation
Writing—original draft	Preparation, creation, and/or presentation of the published work, specifically writing the initial draft (including substantive translation)
Writing— reviewing and editing	Preparation, creation, and/or presentation of the published work by members of original research group, specifically critical review, commentary, or revision, including pre- and post-publication stages

For the analysis of the Q-study, Pearson’s correlation matrix was used, a standardised measure of the linear relationships between two sets of scores. The factor analysis, meaning the identification of patterns and distinct factors, i.e., shared viewpoints, was performed according to principal component analysis (PCA) and a varimax rotation [25]. Selection criteria of the factors considered for further analysis are eigenvalue > 1.0 and at least two Q-sorts loading significantly on the respective factor, with the significance level calculated at p < 0.05. All analyses were conducted using the Q Method Software (https://qmethodsoftware.com/) platform. Crib sheets generated by the Qmethodsoftware.com platform were used for data interpretation to identify the highest- and lowest-ranked statements for each perspective from the statement scores (factor arrays’) [25].

## Results

### CREDIT URE Analysis

Analysis was conducted on 227 matched student and supervisor CREDIT URE ratings, which were based on projects completed across the following clinical specialties and non-clinical disciplines: medicine, surgery, anaesthetics and intensive care medicine, paediatrics, public health, obstetrics and gynaecology, emergency medicine, public health, psychiatry, medical education. Contribution percentages and other summary statistics for the 13 contribution areas are presented in Table 2. Both students and their supervisors most frequently reported students contributing to data collection (i.e. investigation), analysis, curation, and write-up, with over 90% of responses indicating at least moderate involvement in that activity (see Table 2). Another role endorsed by both groups was project management. Less commonly reported roles were the early stages of project development, including conceptualization (ranging from 83.7 to 86.7%) and choice of methodology (ranging from 86.9 to 92.5%). The least common roles involved resources, software, and supervision, and funding acquisition, although a substantial number of students (70–85%) still actively engaged in these activities.

**Table 2:** Summary of CREDIT URE values for both student and supervisor respondents (*N* = 227)

	Students					Supervisors					Cohen’s Ƙ
	% reporting substantive involvement	Mean involvement level	SD	Median involvement level	*N*	% reporting substantive involvement	Mean involvement level	SD	Median involvement level	*N*	
Writing – original draft	97.4	2.95	0.34	3.0	224	98.2	2.93	0.35	3.0	225	0.20
Visualisation	96.0	2.87	0.40	3.0	223	98.2	2.86	0.42	3.0	225	0.25
Data curation	97.7	2.83	0.45	3.0	218	97.4	2.84	0.43	3.0	225	0.20
Formal analysis	97.4	2.83	0.44	3.0	223	96.5	2.82	0.44	3.0	224	0.19
Investigation	94.3	2.78	0.54	3.0	221	96.5	2.76	0.57	3.0	227	0.30
Writing – reviewing and editing	91.6	2.71	0.64	3.0	222	94.9	2.71	0.63	3.0	219	0.20
Project administration	92.3	2.62	0.68	3.0	221	94.7	2.71	0.57	3.0	226	0.17
Validation	88.1	2.39	0.74	3.0	185	92.0	2.63	0.66	3.0	199	0.16
Resources	80.9	2.30	0.94	3.0	194	83.4	2.37	0.94	3.0	210	0.18
Methodology	86.9	2.28	0.76	2.0	222	92.5	2.40	0.71	2.5	226	0.25
Conceptualisation	86.7	2.20	0.67	2.0	225	83.7	2.16	0.72	2.0	227	0.28
Software	70.6	2.07	1.19	3.0	153	85.1	2.46	0.99	3.0	166	0.33
Supervision	73.2	1.95	0.93	2.0	194	76.6	2.28	0.92	3.0	212	0.13

Using the Mann-Whitney U test, students rated themselves lower than the ratings of their project supervisors for the following contributory roles: software (U = 10485.50, z = 2.69, *P* = 0.002, *r* = 0.15); supervision (U = 16054, z = 3.82, *P* < 0.001, *r* = 0.19). Therefore, these Mann–Whitney comparisons showed small effect sizes for both roles. Cohen’s kappa (κ) is a measure of interrater agreement for categorical scales (Cohen, 1960). Across the 13 CREDIT-URE roles, κ values ranged from slight to fair agreement, indicating that while students and supervisors broadly endorsed similar categories of contribution, their judgements of the degree of student responsibility often diverged. Slight agreement (κ ~ 0.01–0.20) suggests limited alignment in perceived responsibility, whereas fair agreement (κ ~ 0.21–0.40) reflects modest but still inconsistent convergence between raters. Table 2 shows fair agreement (0.21–0.40) for eight roles, and slight to none (0.01–0.20) for the remaining five roles.

### Q-methodology Results

25 participants, 16 students (out of *N* = 25 students selected for inclusion, providing a response rate of 64%) and nine project supervisors (out of *N* = 12 supervisors approaches, providing a response rate of 75%), all of whom were affiliated with University College Cork School of Medicine, completed the study. All participating supervisors had previously supervised to completion at least one medical student project. Eleven student participants were in the fourth year of the five-year undergraduate medicine programme, five of them in the third year of the graduate-entry medical programme. Seven medical students had previously participated in a research project prior to the mandatory research project. Supplementary data 2 shows the Q-set statements, with the factor arrays (how a typical student in a factor would rank order the statements). Seven factors with significant factor loading (*P* < 0.05) and eigenvalue > 1.00 (with application of the Kaiser Guttman Criterion; 25) were extracted. Two factors with fewer than two flagged participants were removed, leaving a five-factor solution. The decision to retain a five-factor structure was guided not only by statistical criteria, but also a need for conceptual clarity. The remaining five factors accounted for 55% of the total variance, and each displayed a coherent internal logic based on distinguishing statements. Retaining five factors provided a parsimonious yet sufficiently rich representation of the diverse viewpoints present in the dataset, avoiding over - extraction while ensuring that stable and meaningful student - supervisor perspectives were captured. Table 3 provides detailed Q-sort descriptors for each of the selected factors. Each factor represents a group of participants with similar expectations of student and supervisor roles. With respect to flagged student/supervisor representation across each of the profiles, the following distribution was observed: Profile 1 (6 supervisors, 0 students); Profile 2 (1 supervisor, 3 students); Profile 3 (0 supervisor, 2 students); Profile 4 (0 supervisor, 2 students); Profile 5 (0 supervisor, 2 students). Each factor has a certain number of distinguishing statements that are specific to that factor. The five profiles are summarized in Table 4. The descriptions below provide a narrative interpretation of the relative roles of students and project supervisors across the five profiles, which are informed by Research Skill Development (RSD) Framework [14]. The information in parentheses refers to the specific statement number in the Q-set (between 1 and 99), and its position in the factor array (between − 4 and + 4).

**Table 3:** Q-sorts defining the five factors

Factor/Profile	Loading Q-sorts	Number of Q-sorts	Eigenvalues	% Explained Variance
1	2,3, 8, 13, 22, 25	6	7.93	32
2	6, 14, 16, 24	4	2.06	8
3	12, 17	2	1.51	6
4	11, 20	2	1.15	5
5	10, 19	2	1.06	4

**Table 4:** Summary of the five Q-sort profiles

	Profile 1 Student-driven	Profile 2 Supervisor as guide and overseer	Profile 3 Self-motivated student	Profile 4 Supervisor involved only as needed - skills development	Profile 5 Supervisor as editor
Role of student	The student is expected to play a central role in driving every aspect of the progress, in particular the design and implementation of the data analysis strategy, and all aspects of project management.	The student is expected to be involved in the development of the study question and the choice of methodology.	The student is expected to identify their own skills development needs, as well as independently collect and analyse project data, as well as writing-up study results.	The student is responsible for project progress including all research governance requirements, collection and analysis of data, and adherence with the project delivery timetable.	The role of the student is the implementation of the study design, including data collection, but they are not expected to be completely responsible for data analysis.
Role of project supervisor	The supervisor’s role is particularly focused on providing opportunities for the student to engaged with the wider research community.	The supervisor is involvement in managing the project including securing resources and funding, developing a timetable and monitoring progress, as well as bringing in collaborators.	The supervisor provides a measure of oversight which includes specifying the roles and responsibilities of students/supervisors, and facilitating student access to resources, supports, and provide feedback.	The role of the supervisor involves teaching the students the necessary skills or arranging skill development opportunities for students, including data analysis, technical skills, but not academic writing skills. Otherwise, they should only be available if required.	The supervisor is responsible for setting the research question, provide knowledge of relevant local processes or national approvals, as well as helping the students with the writing of the paper and preparation for publication.

#### Profile 1: Student-driven

This profile explains 32% of the study variance. Six students load significantly onto this factor. Role of student: Students in Profile 1 are expected to be fully immersed in every aspect of the research project. They are expected to plan and design the investigations (17, + 2) and develop awareness of all relevant policies and procedural requirements related to the study (6, + 3). They should be involved in all project management activities including setting deadlines (34, + 3), applying for ethical approval (35, + 4), maintenance of research records (27, + 4). They are responsible for the development of all skills (technical/academic) required for the projects (e.g. 55, + 3) and can be expected to be directly involved in all aspects of project implementation. Role of project supervisor: The supervisor is expected to clarify the roles and responsibility of all others involved in the project (70, + 2), and identify potential collaborators (68, + 2). Otherwise, they are not expected to play an important role in the project.

#### Profile 2: Supervisor as guide and overseer

This profile explains 8% of the study variance. Four students load significantly onto this factor. Role of student: The student is expected to stay on task throughout the project (9, + 3), developing the requisite skills and familiarizing themselves with the relevant literature (2, + 4). They are expected to follow principles of research integrity (24, + 4) while collecting data (18, + 3) and writing up the results (22, + 4). They are also expected to apply for ethical approval (35, + 3), and ensure that deadlines are met (10, + 3). Role of supervisor: The supervisor is expected to lead with respect to selection of the research topic (40, + 3), and the development of appropriate study methodology (42, + 3). The supervisor is also responsible for close oversight of all aspects of project management, including identification of relevant ethical review body (73, + 3), securing funding (77, + 4), and developing contingency plans (50, + 4).

#### Profile 3: Self-motivated student

This profile explains 6% of the study variance. Two students load significantly onto this factor. Role of student: The role of the student is to identify their own skills development needs (25, + 3) and develop skills in those areas necessary for completion of the project (e.g. 15, + 3). They are centrally involved in all aspects of project implementation (e.g. data collection, analysis; e.g. 20, + 3), including day-to-day project management. The student is not involved in higher-order management activities including securing access to services and research facilities (44, −3), and identification of research collaborators (29, −3). Role of project supervisor: The supervisor is principally responsible for senior project management roles including networking and reaching out to necessary collaborators (69, + 4), and facilitating student access to necessary resources (5, + 4). They are also expected to be available at pre-scheduled times for discussion with the student (85, + 3), and for editing of students’ written work (62, 3).

#### Profile 4: Supervisor involved only as needed – skills development

This profile explains 5% of the study variance. Two students load significantly onto this factor. Role of student: The student is expected to assume most of the responsibility for the project and only approach the supervisor when absolutely needed (95, + 4). They are responsible for project progress including all administrative requirements (e.g. 35, + 4), collection and analysis of data (18, + 3), and adherence with the project delivery timetable (10, + 4). Role of project supervisor: The role of the supervisor is to monitor project progress on an ongoing basis (81, + 4), and to be aware of local research policies (45, + 4) and supports available to students (71, + 4). They are also expected to be involved in the early stages of project design planning (56, + 3), and to teach the student the technical skills needed for the project (16, + 4).

#### Profile 5: Supervisor as editor

This profile explains 4% of the study variance. Two students load significantly onto this factor. Role of student: The role of the student is the day-to-day research operations and implementation of the study design including data collection (18, + 4), but not necessarily the analysis (20, −2) or interpretation of results (21, −4). They are also not expected to be involved in formulation of the research question (1, −3). Role of supervisor: The supervisor is expected to provide a research topic (40, + 4) and to oversee the interpretation and write-up of the study results (63, + 4). They are also expected to be familiar with research governance processes.

## Discussion

Medical schools worldwide use a variety of approaches, including active project participation, to promote a better understanding of research methods and how they be used to contribute to patient-centred care. Based on application of the adapted CREDIT URE tool to a sample of 227 capstone medical projects, we noted patterns in relation to contributions and role of students, as well as low-moderate levels of agreement between student and supervisor ratings of level of student involvement across various project activities. Generally, many roles were rated highly both on participation but also regarding level of responsibility. As reported by Honore et al. [22] in a sample of biomedical sciences projects completed in the context of a NIH-funded research training program for undergraduates, the least commonly cited roles were software development and supervision. Both students and their project supervisors rated writing of the original draft article, visualisation (including presentation of published work), data curation, formal analysis, and investigation as the five most common roles and those with the highest level of student responsibility. The prominence of the data curation role is unsurprising as data cleaning and preparation are typically essential elements of traditional clinical research formats including chart or database reviews.

Previous studies involving undergraduate project students have data curation and investigation as the most common roles and those with the highest level of student responsibility [22]. Here, not surprisingly, student involvement in formulation of research goals and aims (i.e. conceptualisation) is rated as less central to the student role than implementation activities. While students may approach clinical supervisors with nascent project ideas, frequently they will be asked to focus on a topic closer to the supervisor’s research interests or clinical activities or interests. In other words, the student may have a part in the process, but the mentor still takes the lead and manages the process. Previous studies have also highlighted that frequent meetings with research supervisors are often needed to discuss the different aspects of this research plan [21]. In a previous study on postgraduate and undergraduate students, this was found to be the most challenging stage in a research course, particularly among undergraduate students [26].

The research roles with the consistently lowest responsibility ratings include supervision (including responsibility for the research activity planning and execution, funding acquisition), and software (i.e., programming and software development). Undergraduate students are usually the least experienced and lowest ranking members of a research lab, so it is not surprising that they are less likely to be engaged in supervision of other research group members. However, over 70% of students reported a degree of supervisory experiences, which may include supervision of secondary level students or early year undergraduates completing summer placements. In other studies employing the CREDIT URE, they reported low student ratings for roles including acquisition of funding, and development of software. In the adapted CREDIT URE employed in the current study, funding acquisition was an omitted role because all medical student projects at our institution need to be either cost neutral or covered by an existing budget. Software development was also one of the least commonly cited roles, mostly because it will not be a feature or requirement for all projects.

In common with many undergraduate programmes, here research supervisors were not specifically offered training associated with project supervision [27, 28]. All had previously supervised more than one undergraduate project previously – in fact all confirmed a minimum of five previously supervised projects, indicative of a surfeit of hand-on experience. These data further support the call for dedicated training around roles and responsibilities of student and supervisors, authorship, communication [21].

The Q-methodology results raises interesting questions regarding the role expectations of medical students and provides insights to approaches towards fostering an improved relationship between students and their research supervisors. There is a consensus across the five profiles that the supervisor’s role should be primarily advisory and concerned with executive tasks, with students doing most of the day-to-day work. However, where there was disagreement across profiles, it related to different expectations around where the line between them is. Members of the ‘Student-drive’ profile expect students to undertake most of the roles; notably, all the members who loaded significantly onto the ‘Student-driven profile are project supervisors. We observed some disagreement over who sets the timeline/deadlines, and who might be responsible for scheduling teaching to ensure that students develop the necessary skills to ensure the project is completed successfully. Based on comparison of the ‘Student-driven’ profile and other available profiles, supervisors generally expected students to fill more roles than students did (see Table 4 for summary of profiles). These results agree with previous quantitative analyses of student expectations around their role in undergraduate research. Using the validated Role Perceptions Rating Scale (RPRS) instrument, in a sample of 320 medical students, Althubaiti & Althubaiti [21] reported that students generally expected the research responsibilities to be equally shared between the supervisor and student during the development and execution of the research project. In particular, students expected the supervisor to be involved during project conceptualisation, ensuring access to facilities, and making a substantive contribution during the project write-up stage.

Research supervisors in the UCC undergraduate medical research programme are primarily clinicians, and they may experience several challenges when participating in research, such as finding adequate time for supervision and availability for their students due to existing clinical commitments [9, 16, 18]. In this context, it is less surprising that they expect students to handle the burden of research governance requirements in addition to all project implementation activities, while their supervision role is more concerned with oversight and introducing students to scholarly networks. These discrepant perspectives on relative project roles of students and supervisors highlight the importance of incorporating a preliminary role expectations assessment (using the CREDIT URE or a similar instrument) at the start of each research project, to ensure a match between what students want to do in a research project, what they expect from their research supervisors, and how adequately supervisors are able to meet their needs [21].

It has been suggested that medical research supervisors need to consider a supervisory approach that is adapted to student-related variables including their level of research experience, as well as factors which relate to the variable and changing needs and requirement of each project stage, from conceptualisation to dissemination [29]. Moller et al. [30] note that some of these issues and discrepancies might be addressed by provision of regular faculty enhancement research workshops are needed to support good research practices, especially for inexperienced research supervisors. In a qualitative analysis of project coordinators’ perceptions of student projects that are successful in terms of developing students’ evidence-based medicine competencies, supervisor availability for the student was cited as one of the major determinants of success, alongside a clear research question and well-defined variables [30]. Student-related determinants included developing ownership and management of the projects, as well as a commitment to developing necessary skills. Compared alongside the five profiles identified Q methodology results, it would suggest that success in terms of achievement of relevant learning outcomes is not aligned with any one of these profiles alone but likely elements of each. Others have noted that as it related to style of supervision, while it will always vary based on supervisor variables and how they adapt their supervision approach to each student, a ‘profile’ which incorporates both providing a scaffold to support students’ learning and fostering the development of autonomy may be a preferred model [30]. These findings offer actionable guidance for practice: fostering early, explicit conversations about supervision style, clarifying expectations for autonomy, and revisiting these agreements as the project evolves. These steps may enhance both learning outcomes and the quality of the supervision experience. Table 5 provides a summary of each of the Q-sort profiles and some implications for staff and students engaged in research projects.

**Table 5:** Summary of Q- sort profiles and practical implications

Profile	Underlying Perspective	Practical Implications for Supervisors	Practical Implications for Students
Student-driven	Student expected to lead most or all aspects of the project	Clarify whether expectations of full autonomy are realistic; ensure foundational skills are present; schedule check-ins	Communicate skills gaps; request guidance early; avoid assuming sole responsibility for complex tasks
Supervisor as guide & overseer	Supervisor leads conceptual and methodological direction; student implements	Provide clear methodological direction; set regular progress reviews; avoid over-directing	Prepare for structured deadlines; build competence gradually; use supervisory guidance effectively
Self-motivated student	Student takes initiative; supervisor supports access and high-level oversight	Ensure access to resources; provide timely feedback; offer academic guidance	Maintain communication; seek help before bottlenecks; use supervisor expertise strategically
Supervisor involved only as needed – skills development	Student leads project but relies on supervisor for targeted skill-building	Identify skill gaps early; schedule teaching sessions; avoid assuming students will cope alone	Identify skills needing support; approach supervisor intentionally; document learning needs early
Supervisor as editor	Supervisor provides topic and major interpretive input	Set expectations for authorship and feedback; avoid fostering dependency	Clarify involvement in write - up; engage actively in interpretation; avoid over -reliance

### This study has limitations

The Q method analysis of student and supervisor views and perspectives is based on a sample with unequal distribution of both groups. Additionally, both analyses were based on a sample of students from a single academic institution, one with a model of undergraduate research participation which is not ubiquitous although an increasing number of medical schools require students to complete mandatory individual projects based in authentic research environments [7, 9].

In summary, these findings highlight several practical implications for the student - supervisor relationship. The divergent profiles identify where expectations around autonomy, supervisory availability, project ownership, and skill development may become misaligned. Clarifying these expectations early, particularly around feedback frequency, research governance tasks, and the level of independence anticipated, may help prevent misunderstandings and support more effective supervision. The results reinforce the value of adaptive supervision, where the level of direction required from supervisors must be adjusted based on the student’s evolving autonomy and readiness. Rather than promoting a single ideal supervisory style, the diverse profiles identified here suggest that effective supervision requires flexibility, responsiveness to student needs, and explicit negotiation of roles.

## Supplementary Information

Below is the link to the electronic supplementary material.

Supplementary Material 1 (DOCX 34.8 KB)

## Data Availability

Data are available on reasonable request.
