# Supplementary material for: Aligning Roles and Responsibilities: Perspectives of Medical Students and Supervisors in Undergraduate Research
Source: Med Sci Educ. 2026 Mar 2;36(3):1247–56. doi: 10.1007/s40670-026-02656-0 (PMC13356107; doi:10.1007/s40670-026-02656-0)
Supplement: Supplementary file 1 — Supplementary Material 1 (DOCX 34.8 KB) [file 40670_2026_2656_MOESM1_ESM.docx]

**Supplementary Data**

Supplementary data 1. Senior Research Project Handbook: The Roles and Responsibilities of Students and Supervisors

Supplementary data 2. Q-set statements and factor arrays (how a typical student in a factor would rank order the statements)

**Supplementary Data 1**

**Senior Research Project Handbook: The Roles and Responsibilities of Students and Supervisors**

The **supervisor** should:

- Understand the Medical School regulations and requirements regarding the senior medical research project
- Know about support services available for the student
- Ensure that s/he has the resources available to see the project to completion
- Discuss the appropriate approval for the project
- Consider the overall feasibility of the project and (where appropriate) have a strategy for modification in the event of major or unforeseen difficulties
- Identify other people who may be involved with the project and clarify their roles and responsibilities
- Introduce the student to others who will be involved in the project
- Offer advice to the student regarding the methodology of the project, although the ultimate decision making, and responsibility lies with the student.

The **supervisor** and the **student** should:

- Agree the direction and extent of the research project including milestones and the responsibilities of the student
- Agree on the accessibility of the supervisor and routes of contact (e.g. scheduled regular meetings, contact policy – text, telephone and e-mail contacts etc.)
- Set a timetable for the stages of the project with agreed milestones and deadlines
- Arrange a feedback process to monitor progress

The **student** should:

- Decide on a project topic and seek a suitable supervisor
- Undertake sufficient background reading to prepare for the project (seeking advice from supervisor if necessary)
- Familiarise themselves with regulations and requirements for the Senior Research Project.
- Consider the necessary time commitment s/he will need to make, and what skills s/he will need to carry out the project
- Complete all project milestones as agreed with supervisor
- Complete and submit project write-up
- Orally present project to examiners on the date specified by the Medical School

**Supplementary Data 2**

| Number | Statement | Factor Array | | | | |
| --- | --- | --- | --- | --- | --- | --- |
|  | | 1 | 2 | 3 | 4 | 5 |
| 1 | The role of the student is to select a research topic | 0 | -3 | 0 | 2 | -3 |
| 2 | The role of the student is to undertake enough background reading to prepare for the project | 3 | 4 | 1 | 2 | 2 |
| 3 | The role of the student is to decide on the appropriate theoretical framework/and or methodology | 1 | -4 | 0 | 1 | -1 |
| 4 | The role of the student is to develop an appropriate timetable for the project | 1 | -2 | 1 | 0 | -2 |
| 5 | The role of the supervisor is to ensure the student has access to the services and facilities required to complete the research | 1 | -1 | 4 | 0 | 3 |
| 6 | The role of the student is to ensure that they are aware of the relevant policies, procedures, and requirements pertaining to the study | 3 | 1 | -2 | -1 | 1 |
| 7 | The role of the student is to schedule meetings between the supervisor and student | 0 | 2 | 2 | 0 | -1 |
| 8 | The role of the student is to coordinate all the communication between the supervisor and the student | 1 | -1 | 1 | 1 | 1 |
| 9 | The role of the student is to ensure that they remain on task | 4 | 3 | 1 | 0 | 3 |
| 10 | The role of the student is to ensure that the project is complete by the submission date | 4 | 3 | 2 | 4 | -2 |
| 11 | The role of the student is to come up with new ideas for alternative research directions, if necessary | 1 | -4 | 0 | 2 | -3 |
| 12 | The role of the student is to set deadlines for the project | 3 | -1 | 0 | -3 | -1 |
| 13 | The role of the student is to overcome any technical issues which may arise | -1 | -2 | -2 | -2 | -4 |
| 14 | The role of the student is to get the research published | -3 | -4 | -1 | -2 | -4 |
| 15 | The role of the student is to develop the student’s academic writing skills | 3 | 3 | 3 | 2 | -4 |
| 16 | The role of the supervisor is to teach the student the technical skills needed for the project | -3 | -2 | -1 | 4 | -1 |
| 17 | The role of the student is to plan and design the investigations | 2 | -1 | 1 | 0 | -2 |
| 18 | The role of the student is to collect the data | 4 | 4 | 4 | 3 | 4 |
| 19 | The role of the student is to decide what types of data analysis to use | 3 | -2 | -2 | -3 | -1 |
| 20 | The role of the student is to analyse the results of the data collection | 4 | 2 | 3 | -1 | -2 |
| 21 | The role of the student is to place the study results in context of the wider literature surrounding the topic of study | 2 | 0 | 1 | 2 | -4 |
| 22 | The role of the student is to write up the results | 4 | 4 | 4 | 4 | 2 |
| 23 | The role of the student is to edit the written work | 3 | -3 | 0 | 1 | -1 |
| 24 | The role of the student is to ensure that the written work follows the rules regarding academic dishonesty and plagiarism | 4 | 4 | 2 | 4 | 1 |
| 25 | The role of the student is to identify the limits of the student’s competence | 1 | 1 | 3 | 3 | 0 |
| 26 | The role of the student is to take detailed notes and record of meetings between the student and supervisor | 3 | 1 | 0 | 1 | 1 |
| 27 | The role of the student is to maintain good records of all research work | 4 | 2 | 1 | 4 | 1 |
| 28 | The role of the student is to consider the overall feasibility of the research | -2 | -4 | -1 | 0 | 2 |
| 29 | The role of the student is to identify other people who may need to be involved with the project | -1 | -3 | -3 | -2 | 0 |
| 30 | The role of the student is to network and reach out to others who may need to be involved in the project | 1 | -4 | -1 | -2 | -2 |
| 31 | The role of the student is to clarify the roles and responsibility of other people who may need to be involved in the project | -2 | -4 | 0 | -1 | -2 |
| 32 | The role of the student is to be aware of support services available for the student | 2 | 0 | 0 | 3 | -1 |
| 33 | The role of the student is to discuss ethical approval for the project | 3 | -1 | -2 | 0 | -3 |
| 34 | The role of the student is to Identify relevant ethical approval documentation required | 3 | -3 | -3 | -2 | 1 |
| 35 | The role of the student is to complete ethical approval applications | 4 | 3 | -2 | 4 | 0 |
| 36 | The role of the student is to Identify and clarify the roles of the student and supervisor | -2 | -2 | -4 | -1 | 2 |
| 37 | The role of the student is to arrange a feedback process to monitor progress | 0 | -2 | 0 | -4 | 2 |
| 38 | The role of the student is to source and obtain any necessary funding for the project | -3 | -4 | -1 | -4 | 1 |
| 39 | The role of the student is to obtain and provide knowledge of relevant local processes or national approvals | 0 | -2 | -2 | 2 | -2 |
| 40 | The role of the supervisor is to select a research topic | -1 | 3 | -2 | -3 | 4 |
| 41 | The role of the supervisor is to undertake enough background reading to prepare for the project | -3 | 0 | -4 | 1 | 0 |
| 42 | The role of the supervisor is to decide on the appropriate theoretical framework/and or methodology | -2 | 3 | 2 | -2 | 2 |
| 43 | The role of the supervisor is to develop an appropriate timetable for the project | -1 | 2 | -3 | -4 | 4 |
| 44 | The role of the student is to ensure the student has access to the services and facilities required to complete the research | -1 | -1 | -3 | -4 | 0 |
| 45 | The role of the supervisor is to ensure that they are aware of the relevant policies, procedures, and requirements pertaining to the study | 0 | 3 | 4 | 4 | 4 |
| 46 | The role of the supervisor is to schedule meetings between the supervisor and student | 0 | -1 | -4 | -4 | 0 |
| 47 | The role of the supervisor is to coordinate all the communication between the supervisor and the student | -3 | -1 | -4 | -3 | 0 |
| 48 | The role of the supervisor is to ensure that they remain on task | -2 | -1 | -3 | 3 | 0 |
| 49 | The role of the supervisor is to ensure that the project is complete by the submission date | -4 | 1 | -4 | -2 | 1 |
| 50 | The role of the supervisor is to come up with new ideas for alternative research directions, if necessary | 1 | 4 | 3 | -1 | 2 |
| 51 | The role of the supervisor is to set deadlines for the project | -1 | 1 | -3 | -1 | -2 |
| 52 | The role of the supervisor is to overcome any technical issues which may arise | -3 | 0 | 2 | 1 | -4 |
| 53 | The role of the supervisor is to get the research published | -4 | 0 | 2 | -2 | 3 |
| 54 | The role of the supervisor is to develop the student’s academic writing skills | -3 | -3 | 3 | -4 | 1 |
| 55 | The role of the student is to develop the technical skills needed for the project | 3 | 2 | -1 | 0 | -4 |
| 56 | The role of the supervisor is to plan and design the investigations | -2 | 0 | 1 | 3 | -1 |
| 57 | The role of the supervisor is to collect the data | -4 | -4 | -4 | -4 | 1 |
| 58 | The role of the supervisor is to decide what types of data analysis to use | -2 | -1 | -2 | -3 | -3 |
| 59 | The role of the supervisor is to analyse the results of the data collection | -4 | -3 | -1 | -4 | -4 |
| 60 | The role of the supervisor is to place the study results in context of the wider literature surrounding the topic of study | -2 | -2 | -2 | 3 | -1 |
| 61 | The role of the supervisor is to write up the results | -4 | -3 | -3 | -4 | -3 |
| 62 | The role of the supervisor is to edit the written work | -3 | 0 | 3 | 1 | 0 |
| 63 | The role of the supervisor is to ensure that the written work follows the rules regarding academic dishonesty and plagiarism | -4 | -3 | -1 | 2 | 4 |
| 64 | The role of the supervisor is to identify the limits of the student’s competence | -3 | 1 | -2 | 0 | -2 |
| 65 | The role of the supervisor is to take detailed notes and record of meetings between the student and supervisor | -4 | -4 | -4 | -4 | 2 |
| 66 | The role of the supervisor is to maintain good records of all research work | -4 | -4 | -4 | 0 | -2 |
| 67 | The role of the supervisor is to consider the overall feasibility of the research | 2 | 3 | 4 | -1 | 4 |
| 68 | The role of the supervisor is to identify other people who may need to be involved with the project | 2 | 3 | 3 | -1 | -4 |
| 69 | The role of the supervisor is to network and reach out to others who may need to be involved in the project | 0 | 2 | 4 | -1 | 3 |
| 70 | The role of the supervisor is to clarify the roles and responsibility of other people who may need to be involved in the project | 2 | 2 | 4 | 3 | 3 |
| 71 | The role of the supervisor is to be aware of support services available for the student | 0 | -2 | 2 | 4 | 4 |
| 72 | The role of the supervisor is to discuss ethical approval for the project | 1 | 1 | 3 | -3 | 2 |
| 73 | The role of the supervisor is to Identify relevant ethical approval documentation required | -2 | 3 | -1 | 2 | 0 |
| 74 | The role of the supervisor is to complete ethical approval applications | -4 | -3 | 0 | -2 | 0 |
| 75 | The role of the supervisor is to Identify and clarify the roles of the student and supervisor | 0 | 4 | -2 | 3 | -1 |
| 76 | The role of the supervisor is to arrange a feedback process to monitor progress | 0 | 2 | -4 | -1 | -1 |
| 77 | The role of the supervisor is to source and obtain any necessary funding for the project | -1 | 4 | 2 | 1 | -1 |
| 78 | The role of the supervisor is to obtain and provide knowledge of relevant local processes or national approvals | -1 | 0 | 0 | 1 | 4 |
| 79 | Student-supervisor relationships are purely professional and personal relationships should not develop | 2 | 1 | -3 | 3 | -3 |
| 80 | The supervisor should insist on regular meetings with the student | 0 | 1 | -4 | -3 | -3 |
| 81 | The supervisor should check regularly that the student is working consistently and is on task | -2 | 0 | -3 | 4 | 0 |
| 82 | The supervisor should be cited as the first or last author on the research paper | -1 | -1 | 2 | -1 | 3 |
| 83 | The student should be cited as the first author on the research paper | -1 | 2 | 4 | -3 | 3 |
| 84 | The supervisor should allow the student to make independent research decisions | 1 | -2 | 1 | 0 | 0 |
| 85 | The supervisor should be available for discussion about the research outside of pre-arranged meeting times | -4 | -2 | 3 | -3 | 3 |
| 86 | The supervisor should be experienced in supervising students | 1 | 1 | 0 | -1 | 2 |
| 87 | The supervisor should be an expert in the project’s research area | 0 | 4 | -3 | 1 | -3 |
| 88 | The supervisor should introduce the student to scholarly networks | -1 | 0 | -1 | 1 | -2 |
| 89 | The student should be allowed to consult other people for expertise and/or skills development | 2 | 2 | 1 | 0 | 1 |
| 90 | The student should be offered constant encouragement, support, and motivation | 2 | 1 | -1 | 3 | 4 |
| 91 | Students should have the opportunity to make mistakes and learn from them | 2 | 0 | 3 | 0 | 4 |
| 92 | The student should be provided an objective assessment of their capabilities regarding the project | -1 | 0 | -1 | 1 | 3 |
| 93 | The supervisor should advocate on the behalf of the student | -3 | 1 | 1 | 2 | 1 |
| 94 | The supervisor should provide references for the student | -2 | -1 | 2 | -3 | -3 |
| 95 | The student should assume most of the responsibility for the project and only approach the supervisor when absolutely needed | 1 | 0 | 1 | 4 | -3 |
| 96 | It is up to the student to ask for constructive criticism from the supervisor | 0 | -3 | 0 | -2 | -4 |
| 97 | It is important that students are engaged and interested in the research | 4 | 2 | 4 | 2 | -4 |
| 98 | It is important for supervisors to be friendly, open, and supportive | 2 | 4 | 4 | -2 | 2 |
| 99 | It is important that students are honest when reporting their progress | 4 | 4 | 2 | 2 | 3 |
